# Investigation of mediastinal lymph node dissection in clinical stage IA pure-solid non-small cell lung cancer patients

**DOI:** 10.1186/s13019-024-02839-z

**Published:** 2024-06-24

**Authors:** Jianlong Bu, Sainan Pang, Xianglong Kong, Benkun Liu, Qifan Xiao, Changfa Qu

**Affiliations:** Department of Thoracic Surgery, Harbin Medical University Cancer Hospital, No.150, Haping Rd., Nangang District, Harbin, Heilongjiang Province 150081 China

**Keywords:** Non-small cell lung cancer, Mediastinal lymph node, Pure-solid nodule

## Abstract

**Objective:**

To explore the independent predictors of pathological mediastinal lymph node (pN2) metastasis in clinical stage IA (cIA) pure-solid non-small cell lung cancer (NSCLC) patients, and to find an appropriate method of mediastinal lymph node dissection.

**Methods:**

This study retrospectively evaluated 533 cIA pure-solid NSCLC patients who underwent radical resection of lung cancer (lobectomy combined with systematic lymph node dissection) from January 2014 to December 2016. The relationship between clinicopathological characteristics and pN2 metastasis was analyzed, and the independent predictors of pN2 metastasis were determined by univariate and multivariate logistic regression analysis. We defined the new factor Y as composed of preoperative cT, CEA, and NSE.

**Results:**

There were 72 cases (13.5%) of pN2 metastasis in cIA pure-solid NSCLC patients. Preoperative clinical tumor diameter (cT), serum CEA level, serum NSE level, and pathological status of station 10 lymph nodes were independent predictors of pN2 metastasis. Patients with cT ≤ 21.5 mm, CEA ≤ 3.85 ng/mL, NSE ≤ 13.40 ng/mL and negative station 10 lymph node group showed lower rates of pN2 metastasis. The new factor Y was an independent predictor of pN2 metastasis. Only 3 (2.1%) of 143 patients in the Y low-risk group showed pN2 metastasis.

**Conclusion:**

For patients with low risk of pN2 metastasis, it might be feasible to take lobe-specific lymph node sampling or systematic lymph node sampling. As for those with high risk of pN2 metastasis, systematic lymph node dissection would be recommended.

## Introduction

Lung cancer is one of the most common malignancies worldwide, with the pathological type being non-small cell lung cancer (NSCLC) in 85% of patients [1]. With the development of radiological techniques, more patients with clinical early-stage NSCLC were diagnosed and treated. Surgery was the most essential treatment for early-stage NSCLC patients, and surgical modalities prefer lobectomy with systematic lymph node dissection (SLND). However, not all NSCLC patients have pathological mediastinal lymph nodes (pN2) metastasis, and whether all early-stage NSCLC patients require SLND remains controversial.

Takizawa et al. reported that the 5-year overall survival rate after surgery was similar between clinical stage I (cI) NSCLC patients undergoing SLND and those who received mediastinal lymph node sampling [2]. Bollen et al. study compared mediastinal lymph node sampling, mediastinal lymph node dissection would increase the complications of surgery, such as recurrent laryngeal nerve injury and lymphatic fistula [3]. Therefore, some scholars support that early-stage NSCLC patients might not need SLND. However, Shentu et al. reported that early-stage NSCLC patients with complete mediastinal lymph node dissection had a better postoperatively overall survival rate compared with mediastinal lymph node sampling [4]. On the other hand, some studies concluded that NSCLC might have skip and multiple metastatic lymph nodes [5]. Thus, SLND was preferred in early-stage lung cancer surgery. Currently, there is no uniform modality for lymph node dissection in early-stage NSCLC patients.

Previous studies have reported that few patients with ground-glass opacity (GGO) based early NSCLC presented with lymph node metastasis [6]. However, relevant studies on pN2 metastasis in clinical stage IA (cIA) pure-solid NSCLC patients have still been limited. Therefore, we aim to find risk factors that could predict pN2 metastasis in cIA pure-solid NSCLC patients, differentiate patient groups with different lymph node metastasis rates, and guide better intraoperative lymph node dissection modalities.

## Methods

### Patients

This retrospective study examined data from NSCLC patients who underwent radical surgery in our center from January 2014 to December 2016. The inclusion criteria were as follows: (1) histopathologically confirmed primary NSCLC; (2) preoperative chest CT showed pure-solid nodules of lung lesions; (3) the patients with cIA NSCLC; (4) blood tests taken 1 week before surgery; (5) surgical method was lobectomy with systematic lymph node dissection; (6) complete clinical, laboratory, and imaging data. Patients were excluded with the following conditions: (1) preoperative chemotherapy and radiotherapy; (2) history of malignant tumor; (3) hematopoietic system or autoimmune disease. Informed written consent was obtained from each patient before the study.

### Clinical data collection

Clinical data including patients’ age, gender, preoperative tumor marker detection results, smoking history, tumor location, clinical tumor diameter, and histopathology were obtained from retrospective electronic medical records. Tumor staging was based on the 8th edition of TNM classification. We combined preoperative factors that predict pN2 metastasis to form a new factor Y.

### Statistical analysis

Mann Whitney U test and chi-square test were applied to the data of cIA pure-solid NSCLC patients to analyze the association between clinicopathological characteristics and pN2 metastasis. The optimal cut-off values for clinical characteristics associated with pN2 metastasis were determined using receiver operating characteristic (ROC) curves and were used to define the high-value group and the low-value group. Univariate and multivariate logistic regression were used to identify independent predictors of pN2 metastasis. SPSS statistics package (SPSS statistics 26.0) was used for all statistical analysis, and *p* < 0.05 was considered statistically significant.

## Results

### Clinicopathological characteristics of stage cIA pure-solid NSCLC patients

533 stage cIA pure-solid NSCLC patients were enrolled in this study, with a mean age of 57.46 ± 8.02 years (range, 29–76 years), and a mean tumor diameter of 21.6 ± 6.0 mm (range, 5–30 mm). Intraoperative pathological types were divided into adenocarcinoma, squamous cell carcinoma, and others. The “others” included 13 patients with pathological types into 6 (1.1%) adenosscale squamous cell carcinomas, 2 (0.4%) large cell carcinomas, 1 (0.2%) carcinoid tumors, 1 (0.2%) poorly differentiated carcinoma, 1 (0.2%) basal cell carcinoma, 1 (0.2%) sarcomatoid carcinoma, and 1 (0.2%) adenoid cystic carcinoma. 72 (13.5%) patients had pN2 metastasis, and the status of pN2 in different lobe tumors is shown in Table 1. A total of 9841 lymph nodes were sampled, with an average of 18.5 ± 6.8 lymph nodes removed per person (range, 9–44), and metastasis pN2 totaled 235. The correlation of clinicopathological characteristics of patients with pN2 metastasis is shown in Table 2.

**Table 1:** The patients with pN2 metastasis in different lung lobes

	Patients of pN2 metastasis (*n*)							
Location (n)	Station 2	Station 3	Station 4	Station 5	Station 6	Station 7	Station 8	Station 9
Right lung lobe (297)	14(4.7%)	9(3.0%)	26 (8.8%)	-	-	20 (6.7%)	1 (0.3%)	2 (0.7%)
Right upper lobe (171)	11 (6.4%)	6 (3.5%)	23 (13.5%)	-	-	7 (4.1%)	1 (0.6%)	2 (1.2%)
Right middle lobe (34)	2 (5.9%)	1 (2.9%)	1 (2.9%)	-	-	4 (11.8%)	0	0
Right lower lobe (92)	1 (1.1%)	2 (2.2%)	2 (2.2%)	-	-	9 (9.8%)	0	0
Left lung lobe (236)	-	-	8 (3.4%)	16 (6.8%)	9 (3.8%)	9 (3.8%)	1 (0.4%)	4 (1.7%)
Left upper lobe (145)	-	-	6 (4.1%)	15 (10.3%)	8 (5.5%)	1(0.7%)	0	0
Left lower lobe (91)	-	-	2 (2.2%)	1 (1.1%)	1 (1.1%)	8 (8.8%)	1 (1.1%)	4 (4.4%)

pN2: pathological mediastinal lymph nodes

**Table 2:** General characteristics and their relationship with pN2 metastasis

		Number (%)/median (range)			
Variables	Total	Negative pN2	Positive pN2	χ^2^/z	*p*
All patients	533	461(86.5%)	72(13.5%)		
Gender				0.495	0.527
Female	283	242(85.5%)	41(14.5%)		
Male	250	219(87.6%)	31(12.4%)		
Smoking history				1.754	0.197
None	325	276(84.9%)	49(15.1%)		
Yes	208	185(88.9%)	23(11.1%)		
Intralobar location				4.997	0.082
Outer 1/3	250	213(85.2%)	37(14.8%)		
Middle 1/3	225	202(89.8%)	23(10.2%)		
Inner 1/3	58	46(79.3%)	12(20.7%)		
Pathological type				2.213	0.340
Adenocarcinoma	428	366(85.5%)	62(14.5%)		
squamous cell carcinoma	92	84(91.3%)	8(8.7%)		
others#	13	11(84.6%)	2(15.4%)		
Tumor locations				1.382	0.850
Right upper lobe	171	144(84.2%)	27(15.8%)		
Right middle lobe	34	30(88.2%)	4(11.8%)		
Right lower lobe	92	80(87.0%)	12(13.0%)		
Left upper lobe	145	126(86.9%)	19(13.1%)		
Left lower lobe	91	81(86.9%)	10(11.0%)		
Station 10 lymph node				60.870	<0.001
Negative	482	435(90.2%)	47(9.8%)		
Positive	51	26(51.0%)	25(49.0%)		
Age (years)		58(29–76)	57(38–75)	-0.805	0.421
Clinical tumor size (mm)		20(5–30)	25(10–30)	-3.170	0.002
CEA (ng/mL)		2.61(0.38–30.68)	3.89(0.72–58.41)	-3.905	<0.001
NSE (ng/mL)		12.70(5.55–31.14)	13.77(7.68–40.38)	-2.507	0.012
CYFRA21-1 (ng/mL)		2.83(0.89–11.56)	2.77(1.29–5.62)	-0.045	0.965
SCCA (ng/mL)		0.80(0.20–16.00)	0.80(0.30–4.10)	-0.726	0.468

Positive pN2: pathological mediastinal lymph nodes metastasis; negative pN2: pathological mediastinal lymph nodes without metastasis; CEA: carcinoembryonic antigen; NSE: neuron-specific enolase; CYFRA21-1: cytokeratin 19 fragment; SCCA: squamous cell carcinoma antigen

### Cut-off values for having relevant clinical characteristics with pN2 metastasis

**Fig. 1:**
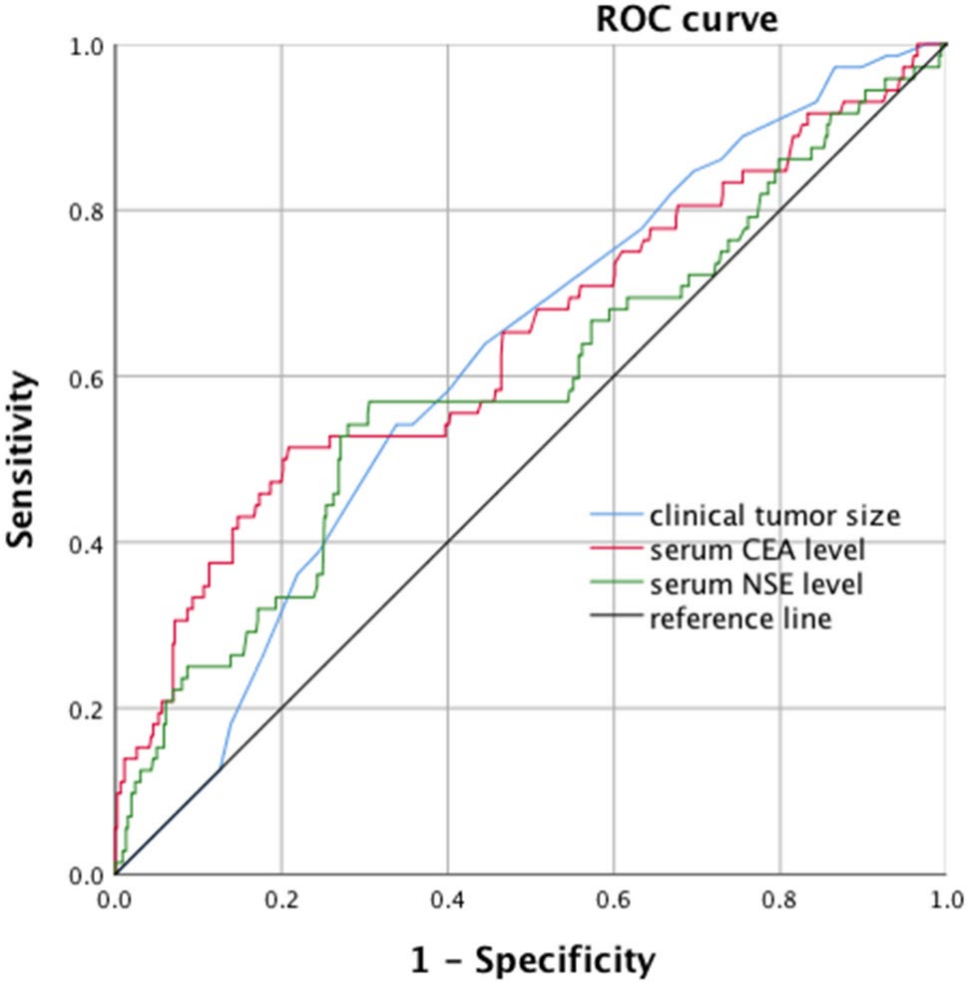
Receiver operating characteristic (ROC) curve of clinical tumor size, serum CEA level and serum NSE level values in predict pN2 metastasis.pN2: pathological mediastinal lymph nodes; CEA: carcinoembryonic antigen; NSE: neuron-specific enolase; The AUC for clinical tumor size was 0.616 (*p* = 0.002, 95%CI: 0.550–0.681) with a sensitivity of 63.9% and specificity of 55.5%; The AUC for serum CEA level was 0.643 (*p <* 0.001, 95%CI: 0.567–0.719) with a sensitivity of 51.4% and specificity of 79.2%; The AUC for serum NSE level was 0.592 (*p =* 0.012, 95%CI: 0.514–0.669) with a sensitivity of 56.9% and specificity of 69.4%

The clinical characteristics with relevance to pN2 metastasis were evaluated by plotting ROC curves, and the optimal cut-off values were determined according to the principle of the maximum of Youden’s index (Fig. 1; Table 3). The optimal cut-off values for clinical tumor diameter (cT), preoperative CEA, and NSE levels were 21.5 mm, 3.85 ng/mL, and 13.40 ng/mL, respectively. The cT > 21.5 mm, CEA > 3.85 ng/mL, and NSE > 13.40 ng/mL groups were prescribed as the high-value group, and the CT ≤ 21.5 mm, CEA ≤ 3.85 ng/mL and NSE ≤ 13.40 ng/mL groups were prescribed as the low-value group.

**Table 3:** Cut-off values of the clinical characteristics in cIA stage NSCLC patients

Variable	Cut-off point	AUC	Sensitivity	Specificity	95% CI	*p*
Clinical tumor size	21.5	0.616	0.639	0.555	0.550–0.681	0.002
Serum CEA level	3.85	0.643	0.514	0.792	0.567–0.719	<0.001
Serum NSE level	13.40	0.592	0.569	0.694	0.514–0.669	0.012

cIA: clinical stage IA; NSCLC: non-small cell lung cancer; AUC: area under the curve; CI: confidence interval; CEA: carcinoembryonic antigen; NSE: neuron-specific enolase

### Analysis of predictive factors for pN2 metastasis

The clinical characteristics from ROC curve analysis and pathological status of station 10 lymph node were used as predictors, and univariate and multivariate logistic regression analyses were performed. cT (*p* = 0.020), CEA (*p* < 0.001), NSE (*p* < 0.001), and pathological status of station 10 lymph node (*p* < 0.001) were independent predictors of pN2 metastasis (Table 4).

**Table 4:** Univariable and multivariable analysis of prognostic factors of cIA stage NSCLC patients with pN2 metastasis

	Univariate			Multivariate		
Variable	Odds ratio	95%CI	*p*	Odds ratio	95%CI	*p*
cT statue	2.209	1.320–3.697	0.003	1.953	1.109–3.438	0.020
CEA statue	4.019	2.404–6.720	< 0.001	2.867	1.634–5.029	<0.001
NSE statue	3.002	1.808–4.983	< 0.001	2.818	1.617–4.911	<0.001
Station 10 lymph node	8.899	4.758–16.644	< 0.001	6.838	3.486–13.412	<0.001

cIA: clinical stage IA; NSCLC: non-small cell lung cancer; pN2: pathological mediastinal lymph nodes; CI: confidence interval; cT: Clinical tumor size; CEA: carcinoembryonic antigen; NSE: neuron-specific enolase

### Effect of combined clinical characteristics in predicting pN2 metastasis

Combined preoperative clinical characteristics that independently predict pN2 metastasis constitute a novel factor Y, we defined patients as Y low-risk group when they had preoperative independent predictors simultaneously as low value (both had cT ≤ 21.5 mm, CEA ≤ 3.85 ng/mL and NSE ≤ 13.40 ng/mL), and the remaining patients as Y high-risk group. A total of 146 patients in the Y low-risk group had 3 (2.1%) presents with pN2 metastasis. A total of 387 patients in the Y high-risk group had 69 (17.8%) patients with pN2 metastasis. By univariate and multivariate logistic regression analysis, both Y (*p* < 0.001) and station 10 lymph node pathologic status (*p* < 0.001) were independent predictors of pN2 metastasis (Table 5).

**Table 5:** Univariate and multivariate logistic regression analysis of Y in predicting pN2 metastasis in cIA stage NSCLC patients

	Univariate			Multivariate		
Variable	Odds ratio	95%CI	*p*	Odds ratio	95%CI	*p*
Y	10.343	3.202–33.413	<0.001	8.977	2.738–29.431	<0.001
Station 10 lymph node	8.899	4.758–16.644	< 0.001	7.813	4.089–14.928	<0.001

NSCLC: non-small cell lung cancer; pN2: pathological mediastinal lymph nodes; Y: New factors in the composition of combined preoperative clinical features; CI: confidence interval

## Discussion

According to the European Society of Thoracic Surgeons (ESTS), mediastinal lymph nodes are processed in 5 ways: (1) selective lymph node biopsy; (2) lymph node sampling, including systemic lymph node sampling (SLNS); (3) systematic lymph node dissection (SLND); (4) lobe-specific lymph node dissection (L-SLND); (5) extended lymph node dissection [7]. For resectable lung cancer, SLND or SLNS was able to provide accurate staging and guide the choice of treatment modality [8]. For the presence of pN2 metastasis in clinical early-stage NSCLC patients, the NCCN also recommends performing SLND or SLNS [9, 10].

The lymph node drainage pattern of stage IA NSCLC was temporarily uncertain whether it follows a lobe-specific pattern, and there were skip and multiple patterns of lymph node metastasis in NSCLC, some scholars believe that SLND remains the preferred modality in the surgical treatment of stage IA NSCLC [5]. However, several studies have shown that SLND for clinical early-stage NSCLC has similar survival to L-SLND and SLNS [2, 11]. Huang et al. suggested that early-stage NSCLC patients who had mediastinal lymph node dissection versus mediastinal lymph node sampling not only had similar overall survival but also similar rates of local recurrence and distant metastasis [12]. ACOSOG Z0030 results from this large prospective clinical trial for patients with pT1-2N0-1 disease who underwent SLND versus those who underwent SLNS showed no difference in recurrence, survival, and surgical complication rates [13]. The results above also indirectly demonstrated that the odds of early-stage NSCLC patients’ pN2 metastasis were smaller.

At present, there is no uniform standard for lymph node resection in early-stage NSCLC, and we tried to search the predictors associated with pN2 metastasis by preoperative clinical characteristics and intraoperative frozen pathology. Cheng et al. showed that the rate of cI NSCLC patients’ pN2 metastasis was 14.2%, and pN2 metastasis was correlated with tumor histology [14]. Yukinori et al. suggested that lymph node metastasis in patients with cIA stage NSCLC is associated with CEA [15]. Previous studies from our center showed that the clinically early-stage NSCLC patients’ lymph node metastasis rate was 6.8%, and the size of solid tumor component and preoperative CEA were independent predictors of lymph node metastasis, which was probably caused by the fact that most patients had ground-glass nodules [16]. Therefore, in this study, we collected data from patients with pure solid NSCLC at stage cIA and found that the rate of pN2 metastasis was 13.5%, and preoperative tumor diameter, CEA, and NSE were independent predictors of pN2 metastasis. Our research results showed that combining three preoperative independent predictive factors to form a new factor Y, pN2 metastasis was shown in only 2.1% (3 patients) of patients in the low-risk group compared with 17.8% (69 patients) of patients in the high-risk group. In this study, the pathological status of the station 10 lymph node was another independent predictor of pN2 metastasis. However, this study used the relevant data of station 10 lymph nodes after operation. If it is used to predict pN2 metastasis, it needs to clarify the situation of all station 10 lymph nodes intraoperatively. At the same time, our results suggested that the station 10 lymph node-negative group (low-risk group) had 47 (9.8%) patients with pN2 metastasis, which might not be an ideal factor to exclude patients without pN2 metastasis. Predictor Y, on the other hand, readily available without increasing the burdens of physicians and patients, might be a better predictor for excluding pN2 metastasis.

Our study found a low rate of lymph node metastasis at stations 8 and 9 in cIA pure-solid NSCLC patients, including 2 patients (0.4%) with station 8 lymph node metastasis and 6 patients (1.1%) with station 9 lymph node metastasis. Asamura and Yang et al. reported at different times that upper lobe lung cancer was prone to superior mediastinal lymph node metastasis; lower lobe lung cancer was prone to inferior mediastinal lymph node metastasis [17, 18]. Lobe-specific lymph node dissection refers to upper lobe lung cancer with resection of the superior mediastinal lymph nodes and lower lobe lung cancer with resection of the subcarinal and inferior mediastinal lymph nodes. Our data suggest that pN2 metastasis largely follows a lobe-specific pattern, with a higher rate of superior mediastinal lymph node metastasis in upper lobe lung cancer; there was a high rate of the subcarinal lymph nodes in lower lobe lung cancer; the right middle lobe lung cancer has a high rate of the subcarinal lymph node metastasis, but also to the superior mediastinal lymph nodes. We believe that the extent of lobe-specific lymph node dissection for middle lobe lung cancer should include the subcarinal and superior mediastinal lymph nodes. Postoperative survival after L-SLND in early-stage NSCLC patients was similar to SLND, and therefore L-SLND was feasible for clinical early-stage NSCLC patients with less pN2 metastasis [11]. The Y low-risk group only had 3 (2.1%) patients who presented pN2 metastasis (station 7 lymph node metastasis in 2 patients with right lower lobe, station 5 and station 6 lymph node metastasis in 1 patient with left upper lobe), and all of the patients were lobe-specific lymph node metastasis. Thus, we considered that lobe-specific lymph node sampling, or SLNS could be adopted for this group of patients. On the other hand, the Y high-risk group had 79 (17.8%) patients with pN2 metastasis, including 15 (3.9%) patients with the presence of non-lobe-specific lymph node metastasis. Thus, patients in the Y high-risk group might benefit from receiving SLND. If intraoperative frozen pathology indicates metastasis in station 10 lymph nodes, SLND would be recommended.

Intraoperative frozen pathology cannot confirm the degree of tumor differentiation and pleural invasion of each specimen in a short time. Thus, the factor cannot be studied as a potential predictor of pN2 metastasis in this test. Pulmonary lymphatic drainage is mainly composed of two major systems, namely interstitial lymphatic vessels and parenchymal lymphatic vessels. There are abundant anastomotic branches between the two major lymphatic drainage systems, and the lymph of lung segments can flow directly to mediastinal lymph nodes [19]. We divided the location of the tumor in the lobe into outer 1 / 3, middle 1 / 3, and inner 1 / 3. The results showed that pN2 metastasis was not correlated with the location of the tumor in the lobe, which did not exclude the correlation with the lymph reflux pathway. The patients included in this study did not have a clear pathological diagnosis of lung tumor before surgery, so the tumor markers related to NSCLC and small cell lung cancer (SCLC) were detected. It is well known that NSE is a relatively specific tumor marker for SCLC. In the course of our study, NSE was found to be an independent predictor of mediastinal lymph nodes in cIA stage pure-solid NSCLC. Previous studies have shown that NSE increases in patients with advanced lung cancer of different pathological types [20]. At the same time, some studies have shown that NSE is associated with the degree of pathological invasion, pathological stage, and prognosis of NSCLC [21, 22]. This phenomenon might be caused by the neuroendocrine characteristics of some patients with NSCLC [23]. More studies are needed to verify whether NSE, a tumor marker of SCLC, is associated with NSCLC.

Our findings suggested a new modality for intraoperative lymph node dissection in cIA pure-solid NSCLC patients, but this study still has some limitations. First, as a single center administrative database, we could hardly capture all the subtle factors, some of which might be critical to the results. Therefore, further multicenter randomized trials are needed to validate these results. Second, in this study, we did not incorporate the relevant data on lymph node micro-metastasis, so it still needs further investigation on whether our findings could apply to patients with lymph node micro-metastasis.

## Conclusions

In the present study, we concluded that preoperative clinical tumor diameter, serum CEA level, serum NSE level, and station 10 lymph node pathologic status are independent predictors of mediastinal lymph node metastasis in cIA pure-solid NSCLC patients. Furthermore, Y, as a novel factor composed of combined cT, CEA, and NSE, is an independent predictor of pN2 metastasis. Meanwhile, the Y low-risk group patients might benefit from receiving lobe-specific lymph node sampling or systematic lymph node sampling, and the Y high-risk group patients might be recommended with systematic lymph node dissection.

## Data Availability

No datasets were generated or analysed during the current study.
